# “A reinstilled hope that they can change”: Facilitator perspectives on a self-care and health promotion peer group program for veterans

**DOI:** 10.3389/fpubh.2022.968281

**Published:** 2023-01-05

**Authors:** Bonnie O. Richard, Melissa H. Abadi, Connor D. Drake, David Rychener, Rachel Bauer

**Affiliations:** ^1^Louisville Center, Pacific Institute for Research and Evaluation, Louisville, KY, United States; ^2^Department of Population Health Sciences, Duke University School of Medicine, Durham, NC, United States; ^3^Durham Center of Innovation to Accelerate Discovery and Practice Transformation, Durham Veterans Affairs Health Care System, Durham, NC, United States

**Keywords:** patient education, peer support, behavior change, veterans, self-care

## Abstract

**Introduction:**

This study assessed the relevance, value, and effectiveness of “Taking Charge of My Life and Health” (TCMLH), a patient wellbeing peer group program for U.S. veterans focused on empowering them to identify what really matters in their lives and to work toward health goals that align with their mission, aspirations, or purpose in life. The potential of TCMLH to empower veterans to engage in self-care behaviors, make health behavior changes, and participate in health care decision making is important, as veterans are more likely than the general population to suffer from multiple chronic conditions that require ongoing self-management.

**Methods:**

We conducted individual semi-structured interviews with 19 TCMLH facilitators serving in eight U.S. Veteran's Health Administration medical centers. Data were analyzed using an inductive approach to identify salient themes in facilitators' experiences.

**Results:**

Facilitators reported that TCMLH participants demonstrated positive attitude changes (e.g., greater confidence and hope) and behavior changes (e.g., making healthcare appointments and implementing self-care practices) by program completion. Further, findings show that mindful awareness practices, the peer group setting, Whole Health assessment tools, and goal setting tools were perceived as the most impactful program elements leading to positive health behavior change.

**Conclusion:**

Overall, findings suggest that this non-clinical peer group program can enhance patient wellbeing, and that there are certain program elements of TCMLH that are driving key attitudinal and behavioral changes.

## Introduction

The U.S. Veteran's Health Administration (VHA), the largest integrated health care system in the country, serves a patient population with higher rates of chronic illness and comorbidities than the general population (1, 2). Health outcomes for patients with common chronic conditions like diabetes and hypertension hinge on patients' ongoing self-care to manage their health conditions (3–5), which often involves learning, implementing, and sustaining substantial behavior changes (5, 6). Patients with chronic conditions have better health outcomes and reduced health care costs if they are more engaged, activated, and empowered—meaning they have knowledge, skills, self-efficacy, and motivation to effectively manage their health needs and health care interactions (7–13). To better serve its patients, the VHA implemented a Whole Health framework, a patient-centered care paradigm wherein veterans' personal values and goals—what patients want their health for—are central to care (14–17). Whole Health emphasizes a comprehensive patient-centered approach to health care that addresses mental, physical, emotional, and social drivers of health in a way that is personalized, proactive, and patient-driven. The goal is to foster greater patient engagement within a system of care that takes a preventive and proactive approach focused on wellbeing rather than one that is reactionary and disease-centric (14–16, 18–20).

The VHA's effort to transform its health care system into one grounded in Whole Health began in 2010 and has since expanded nationwide (15, 17). Implementation of Whole Health involved clinician and staff training, care redesign, cultivating champions, expansion of complementary and alternative health care, and offering aligned patient wellness programs (14, 21–26). The patient wellness program “Taking Charge of My Life and Health” (TCMLH) is a centerpiece for disseminating Whole Health to patients, aiming to foster patient engagement and motivation to make health behavior changes.

TCMLH provides a non-clinical, peer group environment for veterans to learn about Whole Health, the importance of self-care, and ways to achieve values-based health and wellness goals (21).^1^ A standard TCMLH group program involves weekly 90-min meetings over a 2 month period. Drawing from research demonstrating the beneficial impact of peer-based patient education programs on patients' health management, knowledge, and self-efficacy (27–32), learning occurs in a peer group setting that enables shared experience, social support, and accountability. TCMLH is not disease-specific; rather, the program aims to empower participants to identify motivators and provide tools and skills that align with their mission, aspirations, or purpose in life, ultimately bolstering their overall health and wellbeing.

Each TCMLH group program is led by a trained facilitator who is typically also a veteran, and who helps provide a safe, supportive group environment while guiding group participants through an experiential and interactive curriculum. Facilitators, who can be volunteers or VHA staff, go through a didactic and experiential 3-day training, where they learn about Whole Health concepts, tools, and skills and practice facilitating small groups through key session components (21). The facilitator role is designed to foster participants' own efforts in making meaningful health behavior changes—removed from the clinical context of advice-giving and clinical directives. Facilitators do not tell participants what they should be doing or give health recommendations. As Veterans in a given TCMLH group typically have a variety of health challenges, diagnoses, and overall needs, the group is designed to support each individual in taking steps toward improving their self-care and managing their health based on their own reflections on personal values, health and wellness needs, and what efforts are feasible and realistic.

Our multi-component evaluation of TCMLH was part of a larger project to design, implement, and evaluate Whole Health educational and wellness programming throughout the VHA system from 2013 to 2020 (VA777-12-C-002; Rychener, PI). We evaluated the TCMLH program from 2016 to 2018, assessing facilitator trainings (21), site implementation (33), and participant outcomes (34, 35). Our studies of veteran participant outcomes after attending TCMLH found significant improvements from baseline to program conclusion in outcomes related to patient self-management (patient activation, patient motivation, and self-care behaviors) and health indicators (global mental health, perceived stress, and quality of life) (34, 35). Two months post program, participants reported significant improvements in another patient self-management outcome (health care empowerment) and health indicator (global physical health), notably among a sample characterized by very high burden of chronic illnesses (34).

To better understand what may be driving these crucial outcomes, we undertook qualitative research to gain a richer understanding of what, how, and why aspects of TCMLH were beneficial. We conducted individual, semi-structured interviews with experienced TCMLH group program facilitators to explore (a) the most important or valuable aspects of TCMLH in helping veterans implement changes for improved health and wellness, and (b) the changes in attitudes and behaviors endorsed by participants. In this article, we present our findings, focusing on key program elements and their linkages to attitudinal and behavior changes.

## Materials and methods

### “Taking Charge of My Life and Health”

The standard TMCLH program involves 9 weekly in-person 90-min meetings, but has been adapted for completion in shorter time periods, such as 6 weeks or an intensive weekend-long program (21, 34, 35).^2^ Trained facilitators serve as peer guides (not medical experts) for small groups of veterans, and foster a non-judgmental, safe, and supportive environment while guiding veterans through an experiential-heavy curriculum (34, 36). Each meeting includes at least one facilitator-led 5–10-min mindful awareness (MA) practice, such as mindful breathing and guided imagery exercises (36). MA attitudes – including non-judging, being present, having a beginner's mind, and patience – set the framework within which veterans explore their health and wellbeing. MA practices are evidence-based skills to manage stress and promote emotional and psychological wellbeing (37–39).

**Figure 1:**
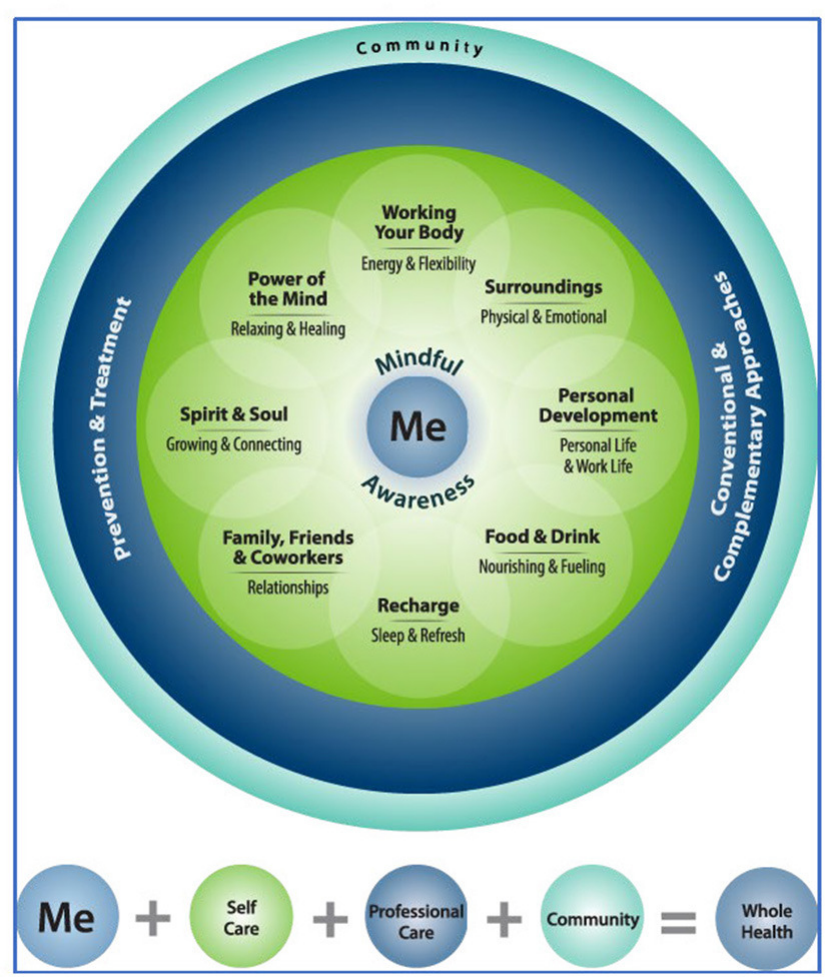
Circle of Health (40).

Throughout TCMLH, participants utilize Whole Health tools – the Circle of Health (Figure 1) and Personal Health Inventory (PHI) – and an action-oriented goal planning strategy to: (a) explore their “Mission, Aspirations, or Purpose;” (b) reflect on their personal values; (c) self-assess health and wellbeing strengths and needs; (d) learn about self-care; (e) set goals; and (f) take action to implement health-related changes (21, 34, 35, 41). The Circle of Health (Figure 1) is a visual touchstone for TCMLH, summarizing how different areas of life are interrelated and may impact one's health (40). The Circle of Health depicts the veteran (“Me”) as central, encircled by the key self-care practice of “Mindful Awareness,” signifying that identifying what matters most starts with reflection and observation. Eight areas of self-care surround the center: Working Your Body; Surroundings; Personal Development; Food and Drink; Recharge; Family, Friends and Coworkers; Spirit and Soul; and Power of The Mind. The outermost rings of the Circle of Health represent the veteran's professional care team and wider community as surrounding supports (14, 40). The second tool, the PHI, is a five-page booklet that operationalizes the eight areas of self-care in the form of a patient self-assessment tool, and is designed to facilitate reflection on areas of needs as well as strengths, and set the course for health behavior change action planning (42). The PHI provides space for veterans to reflect on their values, and then asks them to reflect on and assess each self-care area in terms of where they are now and where they would like to be. The PHI prompts veterans to consider how these assessments connect to their personal motivations and priorities.

Once veterans have determined the self-care area in which they want to focus their efforts, TCMLH participants embark on goal setting. The program uses the “S.M.A.R.T.” goals format, learning to establish a goal that is Specific, Measurable, Action-oriented, Realistic, and Time-specific. Facilitators next guide participants in planning their action steps—thinking through the individual actions that are necessary to make progress toward the goal. Along with goal setting and action planning, TCMLH encourages participants to explore potential barriers to goal achievement and to plan how they can respond to and overcome those barriers. At subsequent sessions, facilitators initiate discussions wherein participants share about their progress as well as challenges they encountered, consider adjustments to their goals or action plans, and gain support from their peers who are doing similar work (36, 41).

### Participants, recruitment, and procedures

Facilitators were eligible to participate in interviews if they had: (a) attended one of 12 facilitator trainings held between 2016 and 2018 that were within the scope of our programmatic evaluation (21), and (b) had facilitated at least one complete TCMLH program at their home VHA site. Considering research on the number of interviews necessary to reach thematic saturation (43, 44), we aimed to recruit 16–20 trained facilitators with varying levels of program delivery experience (i.e., 8–10 facilitators who had implemented fewer than five TCMLH programs and 8–10 who had delivered five or more) (43). The research team recruited facilitators via an email describing the interview purpose, length, topics, and confidentiality procedures. Those who expressed interest were scheduled for phone interviews based on their time and date preferences. As we desired to interview a sample of facilitators with a wide range of experience conducting TCMLH groups, we recruited a convenience sample until we reached saturation in terms of facilitation experience.

At the start of each interview, participants were reminded of the purpose, confidentiality procedures, voluntary nature of interview, and that the interviews would be audio-recorded for transcription. No incentives were provided. The research team was external to the VA, and VA staff did not have access to interview data. The authors' Institutional Review Board deemed this study exempt due to low risk, and the VHA's IRB designated this as a quality improvement evaluation.

###  Interviews

The semi-structured, ~30-min phone interview involved questions about facilitators' observations and experiences with delivering the TCMLH program to veterans. The interview guide was informed by research on health behavior change, and drew on the authors' knowledge of the VA medical system and TCMLH curriculum. To ascertain facilitators' experiences with how TCMLH program components fostered veterans' development of skills, knowledge, and self-efficacy for health behavior change, we asked:

- What Whole Health tools or strategies did you use most often when facilitating the group(s)?
- What skills did you see veterans developing in the program?
- What knowledge do you think the veteran participants gained through the program?
- What would you say was the overall impact/value of TCMLH on the veterans you worked with?
- How would you describe veteran participants at the beginning and end of the program?

To gain broader perspective and fully saturate our data on topics related to potential program impacts, we also asked, ‘How would you describe the role that TCMLH serves within the broader VHA system?' Interviews were conducted in 2018 by BOR, an experienced qualitative researcher, and were audio-recorded.

### Data analysis

The audio files from the interviews were transcribed and de-identified, then uploaded into Dedoose for qualitative analysis (45); all analysis steps were completed by two analysts trained in qualitative analysis methods with knowledge of TCMLH (BOR, RB) and were reviewed by the evaluation lead (MHA). First, transcripts were segmented into domains corresponding with interview topics, allowing for responses from any point in the interview to be grouped with the appropriate topic(s) (46). Next, the team reviewed the content for each interview topic and inductively generated a coding scheme wherein codes were descriptive of content themes (47, 48). Analysts applied codes to text, maintaining intercoder reliability through frequent meetings to review coding, reconcile code application disagreements, hone code definitions, and ensure concept stability. Such processes ensured codes represented concise and mutually exclusive concepts, and that content was comprehensively represented by codes. The combination of (a) interview topics guided by program goals and quantitative findings, (b) content-driven inductive analysis, (c) the iterative process of review and honing, and (d) the analysts' knowledge of TCMLH, provides a strong foundation for authenticity and trustworthiness of our analysis (49).

## Results

Nineteen TCMLH facilitators completed interviews ranging from 23 to 50 min long, averaging 37 min. Facilitators represented eight VA medical systems located throughout the eastern and midwestern U.S. (range 1–5 participants per system). The majority were veterans, and all were VA staff, most commonly in patient-facing non-clinical roles such as Health Coaches and Peer Support Specialists (see Table 1). Those interviewed were evenly split between men and women and averaged 49 years old (range 29–63). Just over half of the facilitators had led five or more TCMLH group programs. Most facilitated nine- or 6-week TCMLH program formats.

**Table 1:** Overview of interview participant characteristics (*n* = 19).

**Characteristic**	***n***	***%***
**Veteran status**
Veteran	15	79%
Non-veteran	4	21%
**Gender**
Men	10	53%
Women	9	47%
**Race**
White	11	58%
Black	5	26%
Other races	2	11%
Race not stated	1	5%
**Ethnicity**
Hispanic/Latino	4	21%
Non-Hispanic/Latino	15	79%
**Education level**
Attended some college	6	32%
Earned college degree (Bachelor's/4-year degree) or higher	13	68%
VHA employee	19^*^	100%
**Job type**
Patient-facing non-clinical role	12	63%
Clinician	3	16%
Administrative role	4	21%
**Number of TCMLH groups facilitated**
1 group	3	16%
2–4 groups	6	32%
5 or more groups	10	53%
**TCMLH format**
9-week program	9	47%
6-week program	7	37%
Other adaptations	3	16%

* Two participants were volunteers when they started as facilitators but had joined the VHA as staff by the time of the interviews.

Facilitators' experiences and perceptions were grouped into two domains: (1) TCMLH program elements most important, beneficial, or impactful for veterans in cultivating health behavior change, and (2) observations of attitude and behavior changes among veterans while participating in TCMLH. Within the first domain, crucial elements of the program that fostered behavior change were (a) MA practices, (b) peer group environment, (c) Whole Health tools—specifically the Circle of Health and Personal Health Inventory (PHI), and (d) the S.M.A.R.T. goal setting process (see Table 2). In the second domain, facilitators described the ways in which they observed veterans change, which were grouped into two broad types: (a) attitudinal improvements (e.g., increased confidence and hopefulness); and (b) improved health behaviors (e.g., addressing health and wellbeing needs with providers, increasing physical activity, and practicing MA) (see Table 3). We present these findings in the following sections, and provide exemplary quotes for each. To preserve anonymity, quotes are not labeled with participant characteristics. To achieve representation across the sample, we reviewed the sources of all quotes to prevent heavy reliance on any one or a few participants, and ensure that quotes provided for each theme came from different participants and that all participants are represented in the results.

**Table 2:** Themes—important/impactful program components of TCMLH.

**Program component**	**Value for participants**
Mindful awareness (MA)	•Develop self-awareness •Manage emotional response, anxiety, stress
Peer group environment	•Positive social support, encouragement •Learning from one another •Accountability balanced with safety of non-judgmental setting
Whole Health tools: - Circle of Health - Personal Health Inventory (PHI)	•Reflect on values and assess health from a whole person perspective •Consider how different areas of life may affect one another •Knowledge of self, including both strengths and weaknesses •Importance of self-care •Form behavior change intentions that are driven by values/motivations
S.M.A.R.T. goal setting	•Form goals that are feasible, realistic •Plan actions necessary to achieve goals •Acknowledge and plan for barriers/challenges •Develops self-efficacy, confidence

**Table 3:** Themes—reported and observed outcomes for TCMLH program participants.

**Change type**	**Outcomes**
Attitudinal	•Greater sense of hope •Self-efficacy in making changes and managing healthcare •Positive self-worth and confidence •More positive view of VA healthcare system
Behavioral	•Started exercising/engaging in physical fitness activities •Active engagement in health care •Practicing MA in daily life •Various other behavior changes: community involvement, actively working on relationships, personal, and professional development, dietary changes

### Most impactful TCMLH program elements on behavior change

#### Mindful awareness practice

Facilitators emphasized that MA practices were a crucial element of the program that had a positive and meaningful impact on participants. According to facilitators, MA practice helped veterans address or improve upon a variety of everyday challenges, including insomnia, strong emotions, and PTSD symptoms, as well as pinpointing sources of increased physical pain during daily activities and identifying other wellness barriers. MA practice also helped veterans find creative ways to improve their environments to support better health, and was a coping strategy for managing stressors and improving communications. Facilitators explained:

“Learning to be more mindful and paying attention more, that has really seemed to hit it with a lot of them, you know, learning to be aware.”

“[MA] helps them to kind of step back, take a breath, and look at things for how they are, maybe not for how their emotions are telling them the way things are. ”

“I've had a lot of veterans just talk about how [MA is] just helping to calm them, just practicing some of the mindfulness skills, you know, they're able to really apply those in a lot of real-life situations.”

“[Program participant] would be in a work environment … with people bringing issues to him that would really just get under his skin. He just adapted doing [an MA practice] … and it just stopped affecting him. He would just rub his thumb and forefinger and remember, like, ‘I don't have to react to this.”'

“From talking to prior group members, they still practice it and I was really surprised by the benefits that they've achieved by practicing mindful awareness.”

#### Peer group environment

Facilitators also asserted that the peer group environment was an essential aspect of the program. Exploring ones' health needs and developing goals took place in the context of a small group of people with similar experiences and challenges, an environment that facilitators were trained to foster as safe and non-judgmental (34). One facilitator described that in the group, “there was this sense of comradery in there...there's a deeper sense of connection with each other.” Participants have unique challenges, health needs, and goals, but they create shared experiences by working through the self-assessment and behavior change processes together. As a facilitator stated, “Where one was lacking, the other was strong, and they helped each other, you know, kind of help figure out their goals and how to best reach their goals. It was really cool to watch.”

The group setting of peers with relatable experiences provided an environment of support, respect, and emotional safety to veterans as they considered their current health and wellbeing, developed goals, and took action. The peer group provided personalized accountability that was attentive to each individual's values and priorities. Veterans enjoyed coming back each week to this meaningful community. Facilitators explained:

“Probably about starting the third or fourth [meeting] they're happy, they want to come... they want to be connected to the other veterans, and by the end they don't want the groups to stop because it's where they're coming to get their support to make healthy changes.”

“I think when they first come in, they don't really know what to expect and they're kind of reluctant... By the end of week two they're comfortable with each other. By week four... they're holding each other accountable, they're asking each other even before class, ‘How are you doing on your goal?”'

#### Whole Health tools

Facilitators asserted that they frequently used and referred back to the Whole Health program tools Circle of Health (Figure 1) and PHI (42), which portray one's overall wellbeing as multi-faceted. Facilitators stated they frequently referenced the Circle of Health to reinforce the concept that these various aspects of one's life are interrelated and influence wellbeing and health. One explained, “They started realizing, ‘Wow, this really does matter to me because I never gave it thought'...they're realizing how much it really does matter.” Further, this tool reinforces self-care as a priority, since it encourages reflection on how they care for themselves could have implications for others: “It helps them to understand that...they need to be the most important person—they need to take care of themselves before they can take care of anyone else.” Facilitators noted that the Circle of Health validates the importance of each participants' chosen goals for their health, even if they are areas of self-care not routinely discussed in a clinical context. For example, a participant knew they needed to improve on both mental and physical health, but by reflecting on the Circle of Health, realized that excess clutter around their home was negatively affecting their mental wellbeing (causing anxiety) and hindering their physical health (there was no space to incorporate a needed home exercise regimen). Thus, the veteran decided to address the “surroundings” area of the Circle of Health, setting a goal around cleaning and organizing their home.

Facilitators also pointed to the importance, value, and relevance of the PHI for veterans in their groups. A facilitator explained, “Everything that they're doing comes back to their PHI, really—what matters to them. …The PHI helps that veteran explore ‘What really matters to me most in this present moment, what am I really ready to tackle right now?”' These realizations helped veterans prioritize and set relevant goals, as well as gain awareness of their strengths and weaknesses. The PHI helps participants gain “self-recognition of their needs as well as their assets, and being comfortable with asking for assistance,” as one facilitator explained. Facilitators said that they could see participants gaining greater understanding of themselves as they worked through the PHI. Further, with the PHI including the eight areas of life from the Circle of Health, it gave them opportunities to choose a place to start that felt more feasible. One facilitator stated: “They're motivated to do the things that they know they can actually do. …They get that extra boost in their self-esteem, you know, ‘OK, I know I can do this; if I start with this, I know I'll have success.”'

#### Goal setting format

Learning an effective goal-achievement process in TCMLH was another crucial element to the program according to facilitators. Participants learned how to set values-based goals, and to plan out actions needed to be successful. Learning how to examine the process for achieving goals was particularly relevant for veterans as their military experience often focused on carrying out orders rather than planning. Goal achievement tools in TCMLH enhanced self-efficacy as participants experienced their progress toward a goal. Facilitators explained:

“As a soldier … we tend to look at the final act, we just charge ahead. … I think when we break that down into small pieces for them, that helps them be able to realize that even some of the goals that they thought were a little bigger might be reachable. … That really helps them a lot.”

“I've had quite a few tell me that they didn't realize that they could do this; these are areas that they thought they would never be able to achieve again. … They understand now that, yes, this is still achievable and with baby steps, that you can have a quality life.”

A component to the goal-setting process in TCMLH is identifying barriers to goal progress and figuring out ways to overcome them. According to facilitators, the concept of planning for how to overcome barriers was new to many participants. As participants worked on taking steps toward their goals during TCMLH, they followed-up each week with their peers and considered how to overcome challenges. The process normalized setbacks, turning the focus away from failure toward learning from these experiences and planning ways to overcome challenges going forward. The attention to identifying and planning for barriers helped veterans stay motivated and build self-efficacy. Facilitators reported:

“A lot of times you hear, ‘Oh, barriers, I didn't even think about barriers.' Like, yep, life happens, there's going to be barriers.”

“Every week I would have them go back and review what their goals were or what went well, what their challenges were—I think that was a vital part. …I think that really was motivating to go, ‘Hey, when I come back next week I want to be able to say I did this or I did that.”'

### Observed attitude and behavior change among veterans

#### Attitude changes

Facilitators observed changes in group participants' attitudes toward themselves, their health, and the VA. They reported that veterans developed hope in themselves, gained self-efficacy in making changes and in managing their healthcare, developed a positive sense of self-worth, increased their confidence, and had a more positive view of the VA. Such changes are important to the VA's goal of providing patient-driven care, which requires patients to be involved as collaborators with their providers and clinical staff. Facilitators described how TCMLH changed attitudes:

“Number one for a lot of them is hope, a re-instilled hope that they can change, hope that maybe the whole VA system and healthcare system is going to shift to better meet their needs.”

“It's that they do have worth and they do have a purpose. It's that they matter to—not just to everybody else that they've been giving to their entire lives—but that they matter to themselves.”

“I have noticed... the ones that have been very... upset with the VA, that those have all been—they're very grateful, they changed to being grateful.”

“[TCMLH] provides veterans with a means of connecting with their provider and then ownership for their health... versus., ‘I'm going to do this because my doctor says I need to do this,' [instead] saying, ‘I'm going to do this because I think it is important to do.”'

#### Behavior changes

Facilitators also described a variety of health and wellbeing behavior changes veterans had undertaken during the group program. Facilitators explained that TCMLH helped veterans re-orient their view of health through identifying their own motivations and priorities for taking health-related action. One facilitator noted he found his role in TCMLH rewarding because it helped participants connect their health needs to their personal motivations in life, saying, “the behavior changes only happen whenever it matters to them.” Facilitators described many participants' implementation of fitness activities, including walking, swimming, and yoga. A facilitator explained,

“Today one of my veterans from one of my first groups stopped by. …He had a new walking partner with him today. And so, not only are they doing the self-care, they're promoting it to their fellow veterans which is wonderful.”

Facilitators reported that veterans in their groups had implemented a variety of health-related behavior changes that covered all areas of the Circle of Health. Prominent examples included stress management with MA or tai chi classes, volunteering in the community, taking steps toward professional development, improving dietary habits, and working on strained relationships.

A vignette illustrates how the program helps veterans start making changes that lead to broader health impacts. In a facilitator's first TCMLH group, a veteran with severe COPD decided that her goal would be to clean and de-clutter her house. As she continued with TCMLH, she soon realized, in the words of the facilitator, “Wait—I've got to de-clutter my *health!*” As she reflected on her complex health challenges with her peers, she realized that the fundamental barrier to progress was her mobility – she had trouble walking and therefore avoided going to healthcare appointments in the expansive medical complex. With the group's support, she pursued eligibility for a power wheelchair and was successful in obtaining one, following which, she embarked on a process of engaging with her providers and learning about her health. The facilitator explained:

“[The power chair] gave her the opportunity to get to some more providers more often and go, ‘Tell me about COPD, tell me about this diagnosis, tell me about that.' She went back to every single one of them and got information about it so she knew. …Now she'll go into the yoga class and park her chair and grab her oxygen and just sit it down and do some yoga.”

This was a veteran who went from not knowing where to start with her cluttered home and complex health challenges, to finding the confidence to seek assistance, increase her knowledge about how to better manage her health conditions, and even try out yoga despite her body's difficulties.

## Discussion

Findings from our facilitator interviews suggest that key program elements were valuable in helping TCMLH participants implement changes to improve their health and wellbeing, and may suggest pathways toward achieving the health outcomes observed in our other studies (34, 35). MA practice was very prominent in facilitators' responses on the value and positive impact of TCMLH for veterans, as well as in the attitude and behavior changes they observed. The growing evidence base on positive outcomes of MA practice includes stress reduction, improved emotional regulation, and decreased experience of negative emotions (37–39, 50–52); facilitators observed similar outcomes and noted they were integral to some of the mental health issues the veterans were experiencing. Indeed, the VA has implemented MA within other aspects of health care delivery, as clinical trials demonstrated significant benefits of MA in decreasing veterans' PTSD and depression symptoms (53, 54). In terms of behavior change, learning MA through practice in each TCMLH session may foster positive outcome expectations and self-efficacy to practice MA on their own (55–60). MA provides veterans with a method through which they can self-monitor, or be more “aware” of their emotions, physical feelings, and external stimuli, which can also help them to sustain other health behavior changes (57–59).

Facilitators' observations of the benefits of the peer group environment also align with research demonstrating the efficacy of peer group programs in promoting self-management and health behavior change for individuals with chronic illnesses (30, 61–66). Studies suggest that being with peers while learning and attempting new or changed behaviors to better address health needs may increase patients' self-efficacy for enacting health promoting behaviors (57, 58, 67, 68). Indeed, facilitators observed that TCMLH participants found common ground in their needs to make changes and in the challenges they faced when attempting to implement changes, and in turn, became supportive resources to one another. Facilitators observed that this environment fostered skill-building and confidence.

Facilitators explained that the Circle of Health and PHI support veterans in realistic self-assessment of their health and help them connect their personal motivations to what aspects of their health they would like to change, leading to development of behavior change intentions. This approach is similar to that of health coaching with its incorporation of motivational interviewing, which can be effective in establishing readiness to make changes for a variety of health concerns (23, 69–74). Focusing on motivations and developing intentions are commonly recognized as important to initial phases of health behavior change, in order to prime the individual toward implementing a new or changed behavior (75). Facilitators noted that group participants tended to view health-related actions as doctors' orders, which left them unmotivated to implement challenging changes in their lives; as such, the time spent in TCMLH reflecting on motivations and the connection of personal values to behavior change intentions was important foundational work for this population.

According to facilitators, setting a S.M.A.R.T. goal in TMCLH and action planning, including planning for challenges, was very effective in spurring veterans toward implementing new or changed behaviors. Research suggests that this type of goal setting is most effective when combined with action planning (76), and TCMLH facilitators' feedback affirmed the benefits of this approach. TCMLH goal setting was effective in helping participants to undertake planned actions, and by making goals feasible, small successes bolstered their confidence.

Many theories of health behavior change emphasize the important role of self-efficacy in undertaking the actions necessary to improve one's own health, which is a recurring outcome of these key program elements according to facilitators (75). The Health Action Process Approach (HAPA) delineates three different types of self-efficacy for behavior change (77), which aligns with facilitators' experiences with TCMLH impacts. These forms of self-efficacy are: “action self-efficacy” (confidence in one's ability to implement the desired change), “maintenance self-efficacy” (confidence in one's ability to overcome barriers), and “recovery self-efficacy” (confidence in one's ability to move forward after experiencing setbacks) (58, 78). In TCMLH, working through the PHI may foster veterans' action self-efficacy; facilitators explained that it helped veterans think of their health more clearly and concretely—as opposed to confronting a complex set of dismaying needs and challenges—and prioritize needs based on their own values. Further, goal setting in TCMLH involves action planning, which also helps participants develop action self-efficacy as small steps seem feasible. Goal setting also involves reflection on challenges and planning how to overcome them, which addresses maintenance self-efficacy; facilitators reported that this was new to many participants and very relevant to their experiences. Finally, reviewing and reflecting on efforts, setbacks, and lessons learned within a supportive group of peers may help participants develop recovery self-efficacy as they can normalize their struggles and encourage one another in moving forward after setbacks (58, 61, 66).

Facilitators observed that TCMLH had positive attitudinal impacts on participants who lacked a sense of hope or had low self-confidence when starting the program. In previous research, we found that at the start of the program TCMLH participants averaged a “very low” score for sense of meaning and purpose in life in comparison to the general population (34). Facilitators reported that veterans gained hope that things can improve, confidence in themselves to make positive health changes and to handle adversity, and felt more empowerment regarding their healthcare. Beyond attitudinal shifts, in our research on patient outcomes, we found that following TCMLH participation, veterans reported statistically significant improvements in measures of self-care behaviors and goal progress (34), which facilitators affirmed as behavioral outcomes they observed in veterans they worked with. Facilitators described a variety of behaviors that participants had undertaken to the benefit of their wellbeing.

Finally, patients' increased involvement in their health care, such as by asking questions of providers to learn about their health conditions, adhering to appointments, and making changes in their daily lives, is crucial for improved outcomes for those with chronic illness. Research underscores the importance for patients with chronic illness to be actively involved in their health care - such patients are more likely to experience better health outcomes and care satisfaction, and have lower health care costs (7, 8, 79–82). Patients' enhanced knowledge and self-management is consistent with the theoretical underpinning of high-quality primary care and chronic disease management, suggesting TCMLH is aligned with clinical efforts to improve health outcomes (12, 83). Further, when providers affirm patients' personal priorities from a whole person perspective, this may encourage patients to improve their self-management, which can ultimately lead to improved health outcomes (13, 82, 84–87). The PHI can help patients communicate with healthcare providers about their personal values and motivations, and the VA has encouraged providers to integrate the PHI in their clinical work with patients. However, it can be difficult for providers to find enough time to engage patients in the discussion and reflection necessary for filling it out during standard appointments (26, 88). TCMLH provides ample time and an ideal environment for introspection and discussion of one's PHI, which they can bring to their next healthcare appointment. In this way, the program can serve as a crucial bridge to providers' efforts to integrate the PHI in patient care. TCMLH is thus an effective mechanism for integrating best practices—such as patient-centered communication, patient engagement, and shared decision-making—to enhance overall wellbeing.

### Limitations

Participation in interviews was voluntary and was not incentivized, thus it is possible that self-selection led to bias toward facilitators who were more enthusiastic about TCMLH. The potential for selection bias in findings is lessened due to the wide range of facilitators' characteristics, including amount of experience facilitating TCMLH. Further, the purpose of the interview as an evaluation component aimed at program improvement was emphasized before and during the interview to discourage social desirability bias. As the evaluation team was external to the VHA, this also provided another degree of separation from facilitators' workplaces.

In most cases, facilitators have limited experience with participants and cannot verify participants' attitudes and behaviors prior to TCMLH; their observations rely on what participants share throughout the program, and in some cases afterwards, as noted in the findings. Additionally, TCMLH participation is voluntary; those who participate through the entirety of the program may be more likely to benefit from it than those who decline to participate or attend infrequently. Facilitators may be more aware of the impacts on veterans who attend regularly and through the entirety of the program because of greater time spent with those veterans. Further, as a VHA program, TCMLH is situated within a large, integrated health care organization providing primary and specialty care, which is connected to further social welfare services and benefits. Such a context may also impact outcomes because program participants all received health care through the same system, perhaps making it easier for some participants to learn to navigate the health system and services through one another's experiences.

This study was conducted before the COVID-19 pandemic which led to alterations in access to patient programming and healthcare, and initiated substantial broadening of access to services via telehealth. Facilitators in this study spoke from their experiences offering the TCMLH group program in person; future research should evaluate implementation and outcomes of TCMLH programs offered remotely via web-based conferencing systems.

## Conclusions

The goal of this study was to gain greater insight into how the TCMLH peer-led group program impacts veteran patient participants, using qualitative data from interviews with experienced TCMLH facilitators, many of whom were also veterans. Facilitator perspectives coalesced around the important roles of MA, the peer group context, the Whole Health tools, and S.M.A.R.T. goal-setting technique in supporting veterans in creating behavior change intentions, gaining self-efficacy, and moving forward with intended actions. Facilitators reported observing and receiving reports from participants of positive changes in attitudes and behaviors, which demonstrated the ways that TCMLH helped participants. Findings reinforce the relevance of the Whole Health paradigm for the U.S. veteran population, which emphasizes the inter-connectedness of different domains of wellbeing, the benefits of self-care, the utility of MA, and the importance of patients' personal values (14, 15, 18, 54, 71). Our findings illuminate mechanisms that may foster the gains observed in physical and mental health, patient motivation, and patient engagement among program participants in our pilot outcomes evaluations (34, 35), and underscore the potential for TCMLH to effectively engage a patient population with a substantial chronic illness burden in health self-management behaviors.

Identification of impactful program elements may be applied to solidifying program fidelity criteria especially given the desire for modified formats (21, 33, 89). This study can be applied to planning future research on program outcomes, including development of measures for various types of outcomes that occur during the process of behavior change, including the different types of self-efficacy, social identification, and empowerment, in addition to outcomes in physical and mental health. Future studies should also consider the broader implementation context (e.g., an integrated healthcare system implementing aligned programming throughout its system) to understand how it may help or hinder outcomes.

## Data availability statement

The raw data supporting the conclusions of this article will be made available by the authors, upon reasonable request.

## Ethics statement

In accordance with U.S. law, The Institutional Review Boards of the Pacific Institute for Research and Evaluation and the Veterans Health Administration deemed this study exempt from human subjects review due to its classification as quality improvement evaluation. As exempt research, written informed consent was not required.

## Author contributions

DR lead development of the Taking Charge of My Life and Health program and facilitator training. MA and CD also contributed to program and training development. MA led program evaluation. Interviews were developed by MA and BR with input from DR. BR conducted interviews and led the analysis. MA, RB, and BR analyzed the interview data, in consultation with DR and CD. BR led manuscript development and writing, with RB, MA, and CD as contributing writers. All authors carefully reviewed the manuscript. All authors contributed to the article and approved the submitted version.

## Footnotes

^1^DR led TCMLH program development and facilitator trainings. MHA and CDD were also involved in the development of TCMLH and pilot facilitator trainings.

^2^For further information about the TCMLH program, facilitator training, and program implementation, see our other publications (21, 33–35).
